# A web-based dynamic nomogram for estimating talaromycosis risk in hospitalized HIV-positive patients

**DOI:** 10.1017/S0950268824001456

**Published:** 2024-12-05

**Authors:** Xu Li, Zhongsheng Jiang, Shenglin Mo, Xiaohong Huang, Tao Chen, Peng Zhang, Linghua Li, Bin Huang, Yanqiu Lu, Ying Wu, Jiaguang Hu

**Affiliations:** 1 Liuzhou Key Laboratory of Infection Disease and Immunology, Liuzhou People’s Hospital, Liuzhou, Guangxi, China; 2 Division of Infectious Diseases, Liuzhou People’s Hospital, Liuzhou, Guangxi, China; 3 Division of Infectious Diseases, The Eighth People’s Hospital of Guangzhou, Guangzhou, Guangdong, China; 4 Division of Infectious Diseases, The Third People’s Hospital of Guilin, Guilin, Guangxi, China; 5 Clinical Research Center, Chongqing Public Health Medical Center, Shapingba, China

**Keywords:** HIV infection, logistic regression, nomogram, predictive model, talaromycosis

## Abstract

Our study aimed to develop and validate a nomogram to assess talaromycosis risk in hospitalized HIV-positive patients. Prediction models were built using data from a multicentre retrospective cohort study in China. On the basis of the inclusion and exclusion criteria, we collected data from 1564 hospitalized HIV-positive patients in four hospitals from 2010 to 2019. Inpatients were randomly assigned to the training or validation group at a 7:3 ratio. To identify the potential risk factors for talaromycosis in HIV-infected patients, univariate and multivariate logistic regression analyses were conducted. Through multivariate logistic regression, we determined ten variables that were independent risk factors for talaromycosis in HIV-infected individuals. A nomogram was developed following the findings of the multivariate logistic regression analysis. For user convenience, a web-based nomogram calculator was also created. The nomogram demonstrated excellent discrimination in both the training and validation groups [area under the ROC curve (AUC) = 0.883 vs. 0.889] and good calibration. The results of the clinical impact curve (CIC) analysis and decision curve analysis (DCA) confirmed the clinical utility of the model. Clinicians will benefit from this simple, practical, and quantitative strategy to predict talaromycosis risk in HIV-infected patients and can implement appropriate interventions accordingly.

Talaromycosis is a systemic infection caused by the dimorphic fungus *Talaromyces marneffei* [1]. HIV-AIDS patients often suffer from talaromycosis, a life-threatening infection that is prevalent mainly in Southeast Asia, India, and South China [2–4]. The HIV epidemic has led to an increase in talaromycosis incidence in recent decades [2, 3]. Currently, the prevalence of HIV-associated talaromycosis in China is approximately 10% [3, 5].

In the absence of timely diagnosis and treatment, talaromycosis is associated with a considerably high mortality rate, which can reach 50% [5–7]. Treatment with antifungal agents during the early stages of talaromycosis can prevent death and significantly reduce the severity of the illness [5, 8–10]. However, targeted interventions can be effective only if individuals at high risk are identified in a timely manner. Currently, the most reliable way to diagnose talaromycosis is through microbiological culture for *T. marneffei*, which can be time-consuming, as it takes approximately ten days to isolate and identify the pathogen from clinical samples, often leading to delays in the initiation of antifungal treatment [3, 11, 12]. Furthermore, certain primary healthcare facilities lack access to expensive diagnostic laboratories for fungus culture. In fact, higher fungal loads are linked to a delay in achieving sterilization [13]. Nevertheless, clinicians must carefully weigh the decision to initiate antifungal therapy in the absence of definitive confirmation of infection. Therefore, efficient, user-friendly, and quantitative assessment tools to evaluate the risk of HIV-associated talaromycosis are needed.

Notably, recent research has shown that nomograms are reliable tools for predicting disease risk and survival rates and play a crucial role in guiding medical treatment [14–16]. A nomogram is a graphical representation utilized in predictive modeling in which various risk factors with different weights are combined to calculate an overall risk score. Consequently, we developed and validated a nomogram for estimating the risk of talaromycosis among HIV-positive individuals. For ease of clinical application, we also developed a web-based nomogram calculator.

## Methods

### Study design

**Figure 1.**
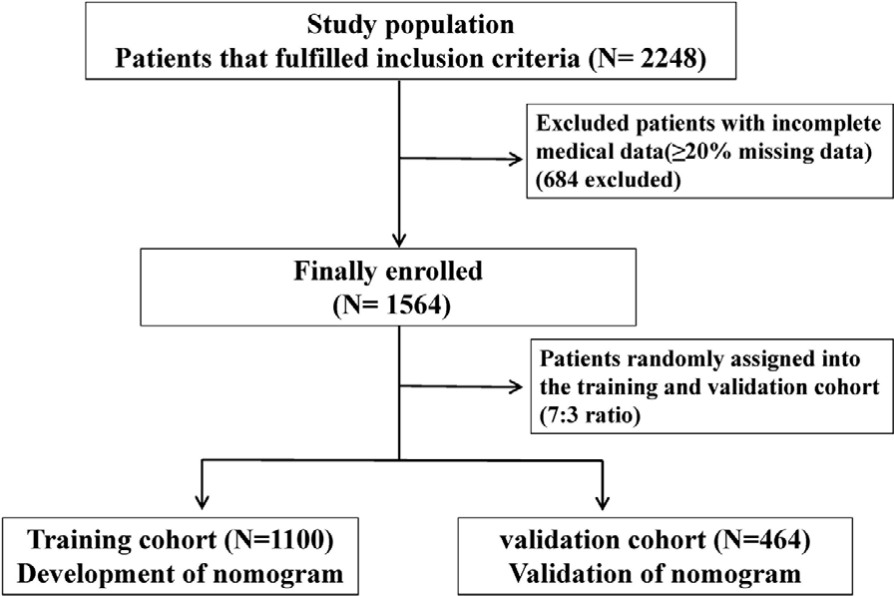
Patient selection flow chart.

The data of the HIV-infected patients analyzed in this study were initially sourced from a retrospective cohort study conducted across multiple medical centres in China. Specifically, the study focused on hospitalized individuals from distinct facilities: Liuzhou People’s Hospital, the Third People’s Hospital of Guilin, the Eighth People’s Hospital of Guangzhou, and Chongqing Public Health Medical Centre. Ethics approval was obtained from the Chongqing Public Health Medical Centre Ethics Committee. Individuals who met the following conditions were included in the study: (1) had a confirmed diagnosis of HIV infection; (2) were aged 18 years or older; (3) had undergone culture and/or histopathological examination for talaromycosis during their hospitalization; and (4) were admitted to the hospital due to suspected opportunistic infections, with a hospital stay lasting more than 3 days. Conversely, individuals who were pregnant or lactating, as well as those with incomplete medical records (≥20% missing data), were excluded from the study. Figure 1 displays the flowchart outlining the criteria for patient inclusion and the data available for analysis.

### Data collection

The data were collected from the medical records of patients who were admitted to hospitals between January 2010 and December 2019. Multiple imputation techniques were used to fill in medical records with incomplete data. The outcome variable of the diagnostic model was defined as whether patients had talaromycosis. Talaromycosis diagnosis was confirmed if (a) *T. marneffei* was isolated from clinical specimens via standard culture methods [3] or (b) *T. marneffei* was discovered in a biopsy tissue sample. Opportunistic infections were described as infections caused by opportunistic pathogens that are typically harmless to individuals with a healthy immune system but can pose a threat to immunocompromised individuals.

The variables considered in our modeling included demographic factors, comorbid illnesses, clinical characteristics, and associated laboratory examination results at admission. Peripheral or abdominal lymphadenopathy (POAL), splenomegaly and hepatomegaly were identified through imaging examinations performed when patients were admitted. The presence of papules, pustules, nodules, subcutaneous abscesses, cysts and ulcers on the skin in patients was documented as skin lesions. The classification of laboratory findings was assigned according to the following criteria: haemoglobin (Hb) level < 90 g/L, defined as a; Hb level ≥ 90 g/L, defined as b; white blood cell (WBC) count = 3.5–9.5 × 10^9^ cells/L, defined as a; WBC count <3.5 × 10^9^ cells/L, defined as b; WBC count >9.5 × 10^9^ cells/L, defined as c; platelet (PLT) count >150 × 10^9^ cells/L, defined as a; PLT count = 100–149.9 × 10^9^ cells/L, defined as b; PLT count = 50–99.9 × 10^9^ cells/L, defined as c; and PLT count <50 × 10^9^ cells/L, defined as d. The thresholds for elevation of aspartate aminotransferase (AST) and alanine aminotransferase (ALT) were set at 40 U/L and 50 U/L, respectively. An AST level > 40 U/L was defined as elevated AST, and an ALT level > 50 U/L was defined as elevated ALT. The AST-to-ALT ratio index (AARI) was determined on the basis of the following categories: (a) ≤1.5, (b) 1.6–3.0, and (c) >3.0. The CD4+ T cell count was classified into three categories: (a) >50 cells/μL, (b) 25–50 cells/μL, and (c) <25 cells/μL.

### Statistical analysis

#### Nomogram development

The remaining HIV-positive patients were stratified into training and validation groups (at a 7:3 ratio) through a random allocation process guided by the predetermined inclusion and exclusion criteria. The comparability of the training and validation groups was subsequently assessed. Logistic regression analysis was employed to ascertain the factors that were independently correlated with talaromycosis. Variables found to be associated with talaromycosis in the univariate analysis (*P* < 0.1) within the training group were included in the multivariate logistic regression analysis to determine the estimated odds ratio (OR) and 95% confidence interval (95%CI). The factors identified as being significantly associated with talaromycosis (*P* < 0.05) in the multivariate logistic regression were utilized to develop a nomogram, which serves as a specific tool for assessing the risk of talaromycosis. The nomogram was constructed by transforming the regression coefficients from the multivariate logistic regression into a 0- to 100-point scale in proportion. The variable with the greatest absolute β coefficient is allocated 100 points to signify its impact. The cumulative points are determined by aggregating the points associated with each independent variable, which are subsequently converted into predicted probabilities.

#### Nomogram evaluation and visualization

The nomogram’s performance was assessed in both the training and validation sets through discrimination, calibration, and clinical usefulness. The prediction nomogram was evaluated for discrimination through the use of ROC curve analysis, from which the AUC, sensitivity, specificity and standard error were calculated. Calibration curves were employed to assess the agreement between the estimated probability and the actual probability, and the accuracy of the nomogram calculation was indicated by how closely the calibration curve aligned with the diagonal line. When the nomogram calibration is accurate, the points should be near the 45-degree line. The clinical efficacy of the nomogram was assessed through decision curve analysis (DCA) and clinical impact curve (CIC) analysis. The creation of the CIC utilized DCA as a framework to visually depict the anticipated number of individuals at elevated risk across different risk categories. To visualize the nomogram, a web-based dynamic application was also developed using the logistic regression model results.

Data analysis was conducted with R version 4.3.0 (http://www.r-project.org) and SPSS version 25.0 (USA, IBM analytics). R packages were utilized for generating visual representations such as nomograms, ROC curves, calibration plots, and DCA graphs. The “DynNom” package was used to dynamically predict talaromycosis risk. All the statistical evaluations were conducted with a two-sided approach. Significant values were determined when the *P* value was less than 0.05.

## Results

### General characteristics

In this study, a total of 2248 HIV-related hospitalized patients meeting the eligibility criteria were initially screened, with 684 subsequently excluded on the basis of predetermined criteria. Finally, 1564 participants were enrolled in the study, with these individuals being divided into a training group (*n* = 1100) and a validation group (*n* = 464) in a 7:3 ratio through random assignment. Table 1 shows the baseline demographic information of the patients in the groups. The characteristics of the two groups were similar, with a marginally significant difference in their distribution (*P* > 0.05).

**Table 1.** Baseline characteristics in training and validation group

Variable	Level	Overall (*n* = 1564)	Training (*n* = 1100)	Validation (*n* = 464)	*P*
TM infection, *N* (%)	No	732 (46.8%)	510 (46.4%)	222 (47.8%)	0.592
	Yes	832 (53.2%)	590 (53.6%)	242 (52.2%)	
Sex, *N* (%)	Male	1172 (74.9%)	815 (74.1%)	357 (76.9%)	0.235
	Female	392 (25.1%)	285 (25.9%)	107 (23.1%)	
Age (years), *N* (%)	<45	611 (39.1%)	419 (38.1%)	192 (41.4%)	0.475
	45–60	506 (32.4%)	361 (32.8%)	145 (31.2%)	
	>60	447 (28.6%)	320 (29.1%)	127 (27.4%)	
Injection drug user, *N* (%)	No	1519 (97.1%)	1073 (97.5%)	446 (96.1%)	0.124
	Yes	45 (2.9%)	27 (2.5%)	18 (3.9%)	
Fever, *N* (%)	No	687 (43.9%)	485 (44.1%)	202 (43.5%)	0.839
	Yes	877 (56.1%)	615 (55.9%)	262 (56.5%)	
Cough, *N* (%)	No	647 (41.4%)	459 (41.7%)	188 (40.5%)	0.657
	Yes	917 (58.6%)	641 (58.3%)	276 (59.5%)	
Skin lesions, *N* (%)	No	1122 (71.7%)	798 (72.5%)	324 (69.8%)	0.276
	Yes	442 (28.3%)	302 (27.5%)	140 (30.2%)	
POAL, *N* (%)	No	1052 (67.3%)	739 (67.2%)	313 (67.5%)	0.916
	Yes	512 (32.7%)	361 (32.8%)	151 (32.5%)	
Hepatomegaly, *N* (%)	No	1310 (83.8%)	921 (83.7%)	389 (83.8%)	0.957
	Yes	254 (16.2%)	179 (16.3%)	75 (16.2%)	
Splenomegaly, *N* (%)	No	1155 (73.8%)	808 (73.5%)	347 (74.8%)	0.585
	Yes	409 (26.2%)	292 (26.5%)	117 (25.2%)	
Oral candidiasis, *N* (%)	No	633 (40.5%)	435 (39.5%)	198 (42.7%)	0.250
	Yes	931 (59.5%)	665 (60.5%)	266 (57.3%)	
Tuberculosis, *N* (%)	No	1373 (87.8%)	965 (87.7%)	408 (87.9%)	0.910
	Yes	191 (12.2%)	135 (12.3%)	56 (12.1%)	
Bacterial pneumonia, *N* (%)	No	1140 (72.9%)	793 (72.1%)	347 (74.8%)	0.274
	Yes	424 (27.1%)	307 (27.9%)	117 (25.2%)	
Hepatitis B, *N* (%)	No	1328 (84.9%)	939 (85.4%)	389 (83.8%)	0.441
	Yes	236 (15.1%)	161 (14.6%)	75 (16.2%)	
Hb (g/L), *N* (%)	≥90	1017 (65.0%)	714 (64.9%)	303 (65.3%)	0.882
	<90	547 (35.0%)	386 (35.1%)	161 (34.7%)	
WBC (10^9^/L), *N* (%)	3.5–9.5	938 (60.0%)	651 (59.2%)	287 (61.9%)	0.265
	<3.5	495 (31.6%)	349 (31.7%)	146 (31.5%)	
	>9.5	131 (8.4%)	100 (9.1%)	31 (6.7%)	
PLT (10^9^/L), *N* (%)	>150	899 (57.5%)	626 (56.9%)	273 (58.8%)	0.376
	100–149.9	251 (16.0%)	170 (15.5%)	81 (17.5%)	
	50–99.9	233 (14.9%)	173 (15.7%)	60 (12.9%)	
	<50	181 (11.6%)	131 (11.9%)	50 (10.8%)	
Elevated ALT, *N* (%)	No	1160 (74.2%)	818 (74.4%)	342 (73.7%)	0.786
	Yes	404 (25.8%)	282 (25.6%)	122 (26.3%)	
Elevated AST, *N* (%)	No	695 (44.4%)	489 (44.5%)	206 (44.4%)	0.983
	Yes	869 (55.6%)	611 (55.5%)	258 (55.6%)	
AARI, *N* (%)	≤1.5	653 (41.8%)	446 (40.5%)	207 (44.6%)	0.266
	1.6–3.0	616 (39.4%)	438 (39.8%)	178 (38.4%)	
	>3.0	295 (18.9%)	216 (19.6%)	79 (17.0%)	
CD4+ T cell count (cells/μL), *N* (%)	>50	362 (23.1%)	252 (22.9%)	110 (23.7%)	0.858
	25–50	319 (20.4%)	222 (20.2%)	97 (20.9%)	
	<25	883 (56.5%)	626 (56.9%)	257 (55.4%)	
Creatinine (mg/L), median (IQR)	NA	69.15 (57.00, 85.00)	69.00 (56.00, 84.43)	70.00 (58.00, 86.00)	0.116
BUN (mmol/L), median (IQR)	NA	4.40 (3.28, 6.18)	4.40 (3.28, 6.18)	4.49 (3.28, 6.20)	0.999

Furthermore, patients in the training group were stratified into two subgroups on the basis of the presence of talaromycosis, as outlined in Table 2. Analysis revealed no statistically significant differences in sex; history of injection drug use; presence of cough, oral candidiasis, tuberculosis, or hepatitis B infection; or creatinine or blood urea nitrogen (BUN) levels between the talaromycosis group (*n* = 590) and the non-talaromycosis group (*n* = 510). Nevertheless, there were notable distinctions in the remaining characteristics between the groups.

**Table 2.** Baseline characteristics of talaromycosis and non-talaromycosis groups

Variable	Level	Overall (*n* = 1100)	Non-talaromycosis (*n* = 510)	Talaromycosis (*n* = 590)	*P*
Sex, *N* (%)	Male	815 (74.1%)	373 (73.1%)	442 (74.9%)	0.502
	Female	285 (25.9%)	137 (26.9%)	148 (25.1%)	
Age (years), *N* (%)	<45	419 (38.1%)	139 (27.3%)	280 (47.5%)	<0.001
	45–60	361 (32.8%)	155 (30.4%)	206 (34.9%)	
	>60	320 (29.1%)	216 (42.4%)	104 (17.6%)	
Injection drug user, *N* (%)	No	1073 (97.5%)	498 (97.6%)	575 (97.5%)	0.840
	Yes	27 (2.5%)	12 (2.4%)	15 (2.5%)	
Fever, *N* (%)	No	485 (44.1%)	266 (52.2%)	219 (37.1%)	<0.001
	Yes	615 (55.9%)	244 (47.8%)	371 (62.9%)	
Cough, *N* (%)	No	459 (41.7%)	199 (39.0%)	260 (44.1%)	0.090
	Yes	641 (58.3%)	311 (61.0%)	330 (55.9%)	
Skin lesions, *N* (%)	No	798 (72.5%)	476 (93.3%)	322 (54.6%)	<0.001
	Yes	302 (27.5%)	34 (6.7%)	268 (45.4%)	
POAL, *N* (%)	No	739 (67.2%)	421 (82.5%)	318 (53.9%)	<0.001
	Yes	361 (32.8%)	89 (17.5%)	272 (46.1%)	
Hepatomegaly, *N* (%)	No	921 (83.7%)	487 (95.5%)	434 (73.6%)	<0.001
	Yes	179 (16.3%)	23 (4.5%)	156 (26.4%)	
Splenomegaly, *N* (%)	No	808 (73.5%)	443 (86.9%)	365 (61.9%)	<0.001
	Yes	292 (26.5%)	67 (13.1%)	225 (38.1%)	
Oral candidiasis, *N* (%)	No	435 (39.5%)	216 (42.4%)	219 (37.1%)	0.077
	Yes	665 (60.5%)	294 (57.6%)	371 (62.9%)	
Tuberculosis, *N* (%)	No	965 (87.7%)	451 (88.4%)	514 (87.1%)	0.508
	Yes	135 (12.3%)	59 (11.6%)	76 (12.9%)	
Bacterial pneumonia, *N* (%)	No	793 (72.1%)	383 (75.1%)	410 (69.5%)	0.039
	Yes	307 (27.9%)	127 (24.9%)	180 (30.5%)	
Hepatitis B, *N* (%)	No	939 (85.4%)	442 (86.7%)	497 (84.2%)	0.256
	Yes	161 (14.6%)	68 (13.3%)	93 (15.8%)	
Hb (g/L), *N* (%)	≥90	714 (64.9%)	389 (76.3%)	325 (55.1%)	<0.001
	<90	386 (35.1%)	121 (23.7%)	265 (44.9%)	
WBC (10^9^/L), *N* (%)	3.5–9.5	651 (59.2%)	336 (65.9%)	315 (53.4%)	<0.001
	<3.5	349 (31.7%)	111 (21.8%)	238 (40.3%)	
	>9.5	100 (9.1%)	63 (12.4%)	37 (6.3%)	
PLT (10^9^/L), *N* (%)	>150	626 (56.9%)	374 (73.3%)	252 (42.7%)	<0.001
	100–149.9	170 (15.5%)	72 (14.1%)	98 (16.6%)	
	50–99.9	173 (15.7%)	41 (8.0%)	132 (22.4%)	
	<50	131 (11.9%)	23 (4.5%)	108 (18.3%)	
Elevated ALT, *N* (%)	No	818 (74.4%)	419 (82.2%)	399 (67.6%)	<0.001
	Yes	282 (25.6%)	91 (17.8%)	191 (32.4%)	
Elevated AST, *N* (%)	No	489 (44.5%)	339 (66.5%)	150 (25.4%)	<0.001
	Yes	611 (55.5%)	171 (33.5%)	440 (74.6%)	
AARI, *N* (%)	≤1.5	446 (40.5%)	261 (51.2%)	185 (31.4%)	<0.001
	1.6–3.0	438 (39.8%)	207 (40.6%)	231 (39.2%)	
	>3.0	216 (19.6%)	42 (8.2%)	174 (29.5%)	
CD4+ T cell count (cells/μL), *N* (%)	>50	252 (22.9%)	166 (32.5%)	86 (14.6%)	<0.001
	25–50	222 (20.2%)	131 (25.7%)	91 (15.4%)	
	<25	626 (56.9%)	213 (41.8%)	413 (70.0%)	
Creatinine (mg/L), median (IQR)	NA	69.00 (56.00, 84.43)	69.05 (56.00, 84.30)	68.90 (56.00, 84.52)	0.961
BUN (mmol/L), median (IQR)	NA	4.40 (3.28, 6.18)	4.49 (3.45, 6.29)	4.30 (3.11, 6.11)	0.130

### Selection of predictors for the construction of a nomogram

In the univariate logistic regression analysis, a total of 16 independent factors were identified as being significantly associated with the risk of talaromycosis in the training set. These risk factors included age, fever, cough, skin lesions, POAL, hepatomegaly, splenomegaly, oral candidiasis, bacterial pneumonia, Hb, WBC, PLT, elevated ALT, elevated AST, AARI, and CD4+ T cell count (*P* < 0.1) (Table 3).

**Table 3.** Univariate and multivariate logistic regression analysis of risk factors of HIV-associated talaromycosis

Variable	Univariate OR (95% CI)	*P*-value	Multivariate OR (95% CI)	*P*-value
Sex				
Male	Reference	Reference	Reference	Reference
Female	0.91 (0.70–1.19)	0.502	/	/
Age (years)				
< 45	Reference	Reference	Reference	Reference
45–60	0.66 (0.49–0.88)	0.005	0.91 (0.61–1.36)	0.649
> 60	0.24 (0.17–0.33)	<0.001	0.38 (0.25–0.58)	<0.001
Injection drug user				
No	Reference	Reference	Reference	Reference
Yes	1.08 (0.50–2.38)	0.840	/	/
Fever				
No	Reference	Reference	Reference	Reference
Yes	1.85 (1.45–2.35)	<0.001	1.06 (0.76–1.47)	0.750
Cough				
No	Reference	Reference	Reference	Reference
Yes	0.81 (0.64–1.03)	0.091	0.78 (0.56–1.08)	0.134
Skin lesions				
No	Reference	Reference	Reference	Reference
Yes	11.65 (8.04–17.37)	<0.001	8.08 (5.09–12.82)	<0.001
POAL				
No	Reference	Reference	Reference	Reference
Yes	4.05 (3.07–5.38)	<0.001	3.04 (2.12–4.37)	<0.001
Hepatomegaly				
No	Reference	Reference	Reference	Reference
Yes	7.61 (4.92–12.31)	<0.001	1.98 (1.10–3.56)	0.024
Splenomegaly				
No	Reference	Reference	Reference	Reference
Yes	4.08 (3.02–5.57)	<0.001	1.10 (0.71–1.71)	0.655
Oral candidiasis				
No	Reference	Reference	Reference	Reference
Yes	1.24 (0.98–1.59)	0.077	0.98 (0.70–1.36)	0.887
Tuberculosis				
No	Reference	Reference	Reference	Reference
Yes	1.13 (0.79–1.63)	0.508	/	/
Bacterial pneumonia				
No	Reference	Reference	Reference	Reference
Yes	1.32 (1.02–1.73)	0.039	1.20 (0.83–1.73)	0.330
Hepatitis B				
No	Reference	Reference	Reference	Reference
Yes	1.22 (0.87–1.71)	0.256	/	/
Hb (g/L)				
≥ 90	Reference	Reference	Reference	Reference
< 90	2.62 (2.02–3.41)	<0.001	1.57 (1.10–2.25)	0.013
WBC (10^9^/L)				
3.5–9.5	Reference	Reference	Reference	Reference
< 3.5	2.29 (1.74–3.01)	<0.001	1.36 (0.94–1.97)	0.108
> 9.5	0.63 (0.40–0.96)	0.035	0.48 (0.27–0.87)	0.016
PLT (10^9^/L)				
> 150	Reference	Reference	Reference	Reference
100–149.9	2.02 (1.43–2.86)	<0.001	1.01 (0.64–1.62)	0.956
50–99.9	4.78 (3.28–7.09)	<0.001	2.06 (1.24–3.44)	0.005
< 50	6.97 (4.40–11.48)	<0.001	2.34 (1.26–4.35)	0.007
Elevated ALT				
No	Reference	Reference	Reference	Reference
Yes	2.20 (1.66–2.94)	<0.001	0.71 (0.45–1.13)	0.147
Elevated AST				
No	Reference	Reference	Reference	Reference
Yes	5.82 (4.49–7.57)	<0.001	3.51 (2.38–5.18)	<0.001
AARI				
≤ 1.5	Reference	Reference	Reference	Reference
1.6–3.0	1.57 (1.21–2.06)	0.001	1.19 (0.83–1.71)	0.339
> 3.0	5.84 (4.01–8.68)	<0.001	2.57 (1.52–4.37)	<0.001
CD4 + T cell count (cells/μL)				
> 50	Reference	Reference	Reference	Reference
25–50	1.34 (0.92–1.95)	0.124	1.35 (0.84–2.20)	0.217
< 25	3.74 (2.76–5.11)	<0.001	2.52 (1.67–3.80)	<0.001
Creatinine (mg/L)	1.00 (1.00–1.00)	0.773	/	/
BUN (mmol/L)	0.99 (0.96–1.01)	0.259	/	/

Multivariate logistic regression models were subsequently constructed by integrating the aforementioned 16 variables. The outcomes of the logistic regression analysis are presented in Table 3, revealing a significant decrease in talaromycosis risk among elderly patients (age > 60 years) [OR: 0.38; 95% CI (0.25–0.58)] or in patients with higher WBC counts (>9.5 × 10^9^ cells) [OR: 0.48; 95% CI (0.27–0.87)]. Importantly, the presence of skin lesions had a stronger association with the likelihood of talaromycosis [OR: 8.08; 95% CI (5.09–12.82)]. Patients who presented with POAL or hepatomegaly presented an increased risk of talaromycosis, with OR of 3.04 (95% CI 2.12–4.37) and 1.98 (95% CI 1.10–3.56), respectively. Additionally, patients who exhibited a decreased Hb level (<90 g/L), decreased PLT count (<100 × 10^9^ cells), elevated WBC count (>9.5 × 10^9^ cells), elevated AST, increased AARI (>3), or decreased CD4+ T cell count (<25 cells/μL) were also found to have increased susceptibility to talaromycosis (*P* < 0.05).

### Nomogram development, evaluation and visualization

**Figure 2.**
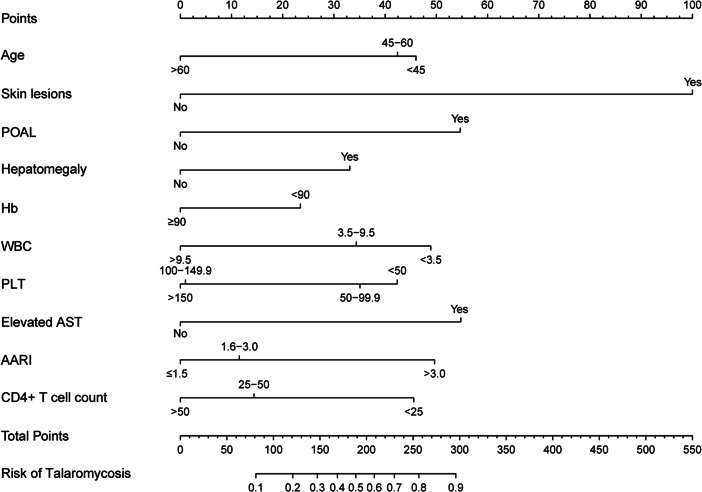
A nomogram for the diagnosis of HIV-associated talaromycosis. The nomogram requires adding the points from each individual factor to calculate the total points and then drawing a vertical line on the total points to determine the corresponding risk level for talaromycosis.

On the basis of the findings of the multivariate logistic regression analysis, a risk prediction nomogram (Figure 2) was developed using independent predictors. The probability of talaromycosis can be ascertained by aggregating the designated points for each variable in the nomogram. The total score was subsequently translated into an individual risk assessment for talaromycosis. A higher cumulative score was indicative of an increased risk of talaromycosis.

**Figure 3.**
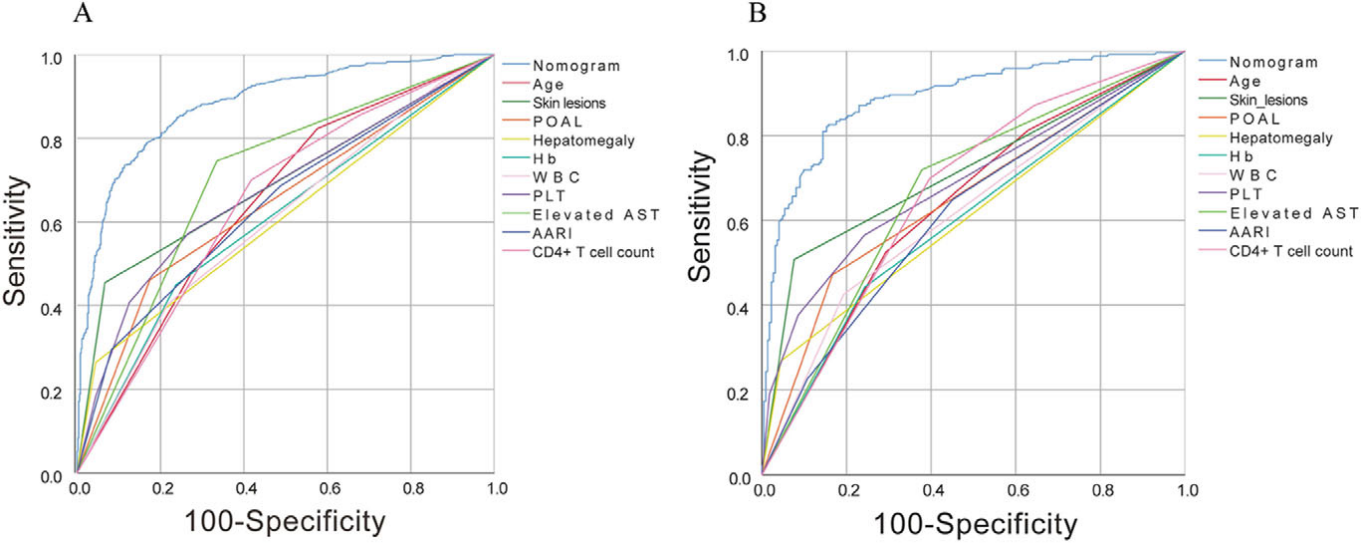
ROC curves were generated for the nomogram to predict talaromycosis risk in both the training (A) and validation (B) groups.

To evaluate the predictive performance of the models, ROC curves were generated for both the training and validation groups (Figure 3). Table 4 presents the sensitivity, specificity, standard error, and AUC values of the models. ROC curve analysis demonstrated that the AUC of the nomogram outperformed any single factor in both the training and validation groups. The AUC values for the prediction nomogram in the training and validation groups were 0.883 (95% CI: 0.863–0.903) and 0.889 (95% CI: 0.859–0.919), respectively, indicating strong discriminatory and predictive capabilities of the model.

**Table 4.** Model evaluation metrics in training and validation groups

	Training group				Validation group			
Variable	AUC (95% CI)	S.E	Sensitivity	Specificity	AUC (95% CI)	S.E	Sensitivity	Specificity
Nomogram	0.883(0.863–0.903)	0.010	79.0%	83.3%	0.889(0.859–0.919)	0.015	82.2%	84.7%
Age	0.648(0.615–0.681)	0.017	82.4%	42.4%	0.638(0.588–0.688)	0.026	52.5%	70.7%
Skin lesions	0.694(0.663–0.725)	0.016	45.4%	93.3%	0.716(0.669–0.763)	0.024	50.8%	92.3%
POAL	0.643(0.611–0.676)	0.017	46.1%	82.5%	0.652(0.602–0.702)	0.025	47.1%	83.3%
Hepatomegaly	0.610(0.577–0.643)	0.017	26.4%	95.5%	0.612(0.561–0.663)	0.026	26.9%	95.5%
Hb	0.606(0.573–0.639)	0.017	44.9%	76.3%	0.599(0.548–0.651)	0.026	44.2%	75.7%
WBC	0.605(0.572–0.638)	0.017	40.3%	78.2%	0.621(0.570–0.671)	0.026	42.6%	80.6%
PLT	0.674(0.642–0.705)	0.016	57.3%	73.3%	0.688(0.640–0.736)	0.025	56.6%	75.7%
Elevated AST	0.705(0.674–0.737)	0.016	74.6%	66.5%	0.670(0.621–0.720)	0.025	71.9%	62.2%
AARI	0.643(0.611–0.675)	0.016	29.5%	91.8%	0.615(0.564–0.666)	0.026	64.9%	55.0%
CD4+ T cell count	0.648(0.615–0.680)	0.017	70.0%	58.2%	0.666(0.616–0.716)	0.025	69.8%	60.4%

AUC, area under the curve; 95%CI, 95% Confidence interval; S.E, Standard error.

**Figure 4.**
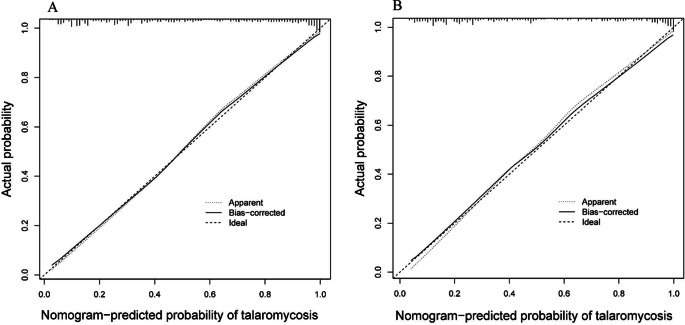
Calibration curves for the nomogram model in the training set (A) and validation set (B). The performance of the nomogram is represented by a dashed line. The dashed line serves as a reference for where an ideal nomogram should be located, while the solid line is an adjustment for potential bias within the nomogram.

**Figure 5.**
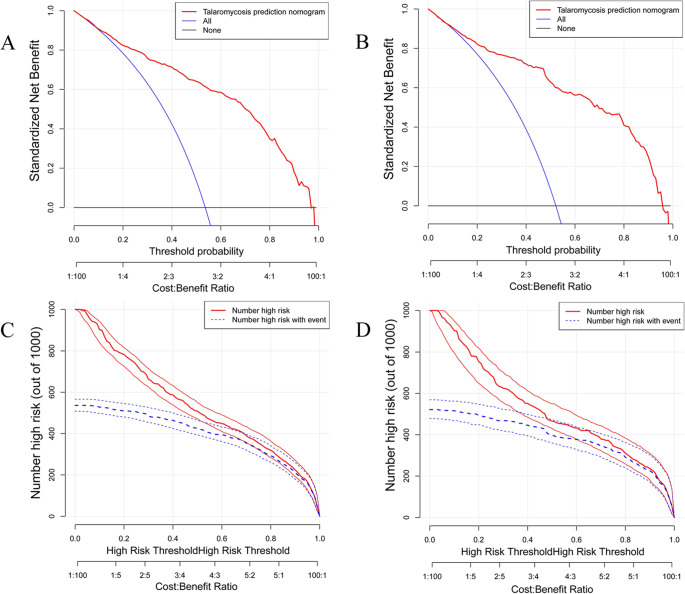
The nomogram decision curve (A, B) and clinical implications (C, D) for assessing the risk of talaromycosis in both the training and validation sets are presented. The red line (number at high risk) represents the number of individuals classified as positive (high-risk individuals) by the nomogram at various threshold probabilities, whereas the blue line (number of individuals at classified as positive) represents the number of true positives at each threshold probability.

Furthermore, calibration curves were constructed for both the training and validation datasets, demonstrating the concordance between the predictive probability of the nomogram and the observed outcomes for talaromycosis. The close proximity of the calibration plot points to the 45-degree line suggests robust calibration of the nomogram model (Figure 4). Next, DCA was employed to assess the practicality of the nomogram in guiding decision-making (Figure 5). The DCA indicated that utilizing the nomogram for predicting talaromycosis provided greater benefit than either treating all patients or treating none when the threshold probability of a patient fell within the range of 3–97% in the training groups. Furthermore, the concordance index of the nomogram indicated that in the optimal probability range, the patients predicted to be at high risk consistently outnumbered the actual patients with talaromycosis while maintaining a satisfactory cost–benefit ratio. The favourable clinical utility of the nomogram was also demonstrated in the validation group on the basis of the results of DCA and CIC analysis (Figure 5).

**Figure 6.**
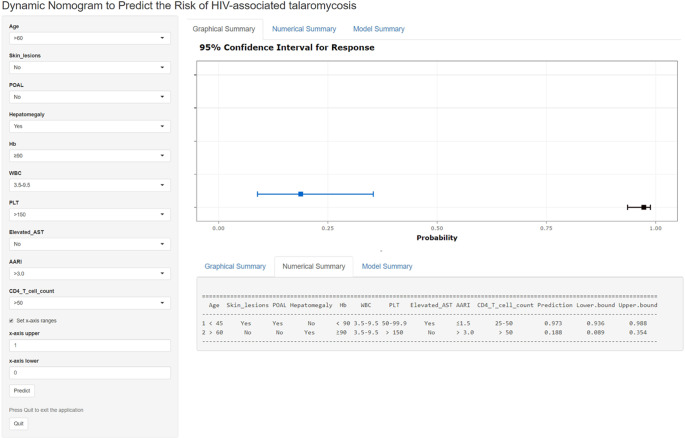
A web-based dynamic nomogram was developed for the prediction of talaromycosis. By inputting a participant’s clinical variables into the online tool at https://lzry-talaromycosis.shinyapps.io/DynNomapp/, the corresponding probability of developing talaromycosis can be obtained. The figure shows that the probabilities of talaromycosis development for the two patients in our study were 0.188 (95% CI: 0.089–0.354) and 0.973 (95% CI: 0.936–0.988).

Additionally, to increase the accessibility of the nomogram for healthcare professionals, a web-based interactive application (https://lzry-talaromycosis.shinyapps.io/DynNomapp/) was created. This tool is readily available online and can be utilized freely by the public. Through this application, clinicians can promptly determine a patient’s likelihood of developing talaromycosis by inputting relevant clinical variables and reviewing the output results provided by the website (Figure 6).

## Discussion

Approximately 50000 people with HIV are believed to contract talaromycosis annually in regions with high risk [17]. Delayed identification is the primary cause of the elevated mortality rate in HIV-positive individuals coinfected with *T. marneffei*, with numerous instances of talaromycosis remaining unnoticed until advanced phases [12, 18, 19]. To address this issue, we pinpointed ten potential risk factors for HIV-related talaromycosis and created a nomogram along with a user-friendly online tool using the established model. The nomogram created in this research functions as a valuable instrument for evaluating the likelihood of talaromycosis, enabling the distinction between individuals with a heightened risk and those with a lower risk to some degree.

Skin lesions can manifest as the initial or solitary symptom of *T. marneffei* infection [5]. This study revealed that 45.4% of talaromycosis-infected patients exhibited skin lesions, a prevalence consistent with findings from northern Thailand (40.7%) and eastern China (43.8%) [12, 20]. In the context of multivariate logistic analysis, skin lesions emerged as a significant risk factor for talaromycosis. Clinicians may consider a tentative diagnosis of talaromycosis when patients present with skin lesions [2, 5]. Thus, the presence of skin lesions may be an indication for a more expedited process for obtaining blood cultures for diagnostic purposes and initiating antifungal therapy, resulting in a shorter mean duration of antifungal treatment and improved clinical outcomes [18]. Nevertheless, the underlying pathogenesis and prognosis of skin involvement remain inadequately understood.

Furthermore, individuals with elevated AST levels and AARIs were at greater risk of developing talaromycosis. AST and ALT are crucial serum biomarkers for evaluating liver damage, with each being responsive to injury to mitochondria and hepatocyte membranes [21]. Our research revealed that 73.79% of individuals with talaromycosis had elevated AST levels, which is consistent with the findings of previous studies [5, 22]. Moreover, there has been an increase in the correlation between the AARI and the risk of talaromycosis in patients. In fact, previous research has demonstrated an increased AARI in individuals with talaromycosis [18, 23]. An increased AARI is significantly correlated with mortality rates in patients with talaromycosis [24]. Additionally, the findings of research on fungal diseases have suggested that the mean AARI in patients with disseminated histoplasmosis exceeds that of those with localized pulmonary disease and other mycoses [25]. Therefore, elevated AST and AARI levels could support the identification of talaromycosis.

Consistent with findings from other studies, individuals with HIV infection who were afflicted with talaromycosis tended to be younger and exhibit severe immunosuppression, as evidenced by low CD4+ T cell counts [7, 12, 22]. In our study, most HIV-infected patients with talaromycosis had hepatomegaly and POAL. Clinically, talaromycosis is defined by the invasion of various organ systems by fungi, which can grow in macrophages, and infection can spread through the reticuloendothelial system [3]. Previous studies have shown that clinical symptoms (e.g., hepatomegaly and lymphadenopathy) are commonly observed in the disease course [12, 18, 22, 26]. Additionally, Hb levels, WBC counts, and PLT counts are essential for model prediction. The lower the Hb level, WBC count or PLT count are, the greater the risk of talaromycosis. Indeed, this phenomenon has also been observed in other studies [12, 18, 22, 26]. Hence, clinicians should remain vigilant for potential mixed infections involving talaromycosis in patients with low CD4+ T cell counts, particularly in endemic regions, even in the absence of skin lesions. This vigilance is especially important when patients present with an elevated AST level and AARI; symptoms of POAL and hepatomegaly; and a low Hb level, WBC count, or PLT count.

Nomograms serve as valuable risk prediction tools in medical practice and are commonly utilized for diagnosing medical conditions and assessing clinical outcomes [27, 28]. In the context of this study, the nomogram specifically designed for talaromycosis risk assessment effectively differentiates between patients at high and those at low risk. Internal validation of the nomogram revealed strong discrimination and calibration abilities, whereas the results of additional analyses, such as DCA and CIC analysis, further supported the clinical utility and performance of the nomogram. Ultimately, a dynamic web-based calculator was created to streamline the application process. Our nomogram model revealed that patients at high risk for talaromycosis can be accurately identified for increased postoperative monitoring and prompt medical intervention, whereas unnecessary antifungal treatment can be avoided for patients at low risk, thereby reducing both pharmaceutical and economic costs. This tool will assist clinicians in making more informed decisions.

This study has several limitations that should be acknowledged. First, owing to the retrospective nature of the data collection in this study, serological fungal markers such as (1–3)-β-D glucan and galactomannan were excluded from the analysis because the missing data rate exceeded 20%, and the evidence level may therefore be lower than that in a prospective study. Second, the applicability of this prediction model may be limited in settings with lower talaromycosis prevalence rates, thus restricting the generalizability of the findings. Finally, this research was carried out utilizing data solely from patients of Chinese descent. The generalizability of our findings to populations in different geographical regions remains uncertain. Additional research is necessary to explore this topic in non-Chinese populations.

Despite the aforementioned limitations, this multicentre, retrospective study included individuals from both endemic and nonendemic regions of China. Through analysis of basic demographic and nonspecific laboratory indicators, our study has yielded a prediction nomogram that can be readily utilized to evaluate the risk of talaromycosis in HIV-infected individuals.

## Conclusions

In conclusion, a cost-effective interactive nomogram was created to estimate the risk of talaromycosis utilizing pertinent data gathered from patients in various hospitals. By combining ROC curves, calibration curves, DCA, CIC, and a web calculator, we developed a nomogram that precisely evaluates an individual’s talaromycosis risk, aiding clinicians in their decision-making process.

## Data Availability

The data utilized in this research can be obtained from the corresponding author upon a justifiable request.
